# Heteroatom-Doped Carbon Nanostructures Derived from Conjugated Polymers for Energy Applications

**DOI:** 10.3390/polym8100366

**Published:** 2016-10-17

**Authors:** Yanzhen He, Xijiang Han, Yunchen Du, Bin Zhang, Ping Xu

**Affiliations:** School of Chemistry and Chemical Engineering, Harbin Institute of Technology, Harbin 150001, China; heyanzhen2015@126.com (Y.H.); yunchendu@hit.edu.cn (Y.D.); zhangbin_hit@aliyun.com (B.Z.)

**Keywords:** conjugated polymers, heteroatom-doped carbon, fuel cells, supercapacitors, batteries

## Abstract

Heteroatom-doped carbon materials have been one of the most remarkable families of materials with promising applications in fuel cells, supercapacitors, and batteries. Among them, conjugated polymer (CP)-derived heteroatom-doped carbon materials exhibit remarkable electrochemical performances because the heteroatoms can be preserved at a relatively high content and keep stable under harsh working conditions. In this review, we summarized recent advances in the rational design and various applications of CP-derived heteroatom-doped carbon materials, including polyaniline (PANI), polypyrrole (PPy), and their ramification-derived carbons, as well as transition metal-carbon nanocomposites. The key point of considering CP-derived heteroatom-doped carbon materials as important candidates of electrode materials is that CPs contain only nonmetallic elements and some key heteroatoms in their backbones which provide great chances for the synthesis of metal-free heteroatom-doped carbon nanostructures. The presented examples in this review will provide new insights in designing and optimizing heteroatom-doped carbon materials for the development of anode and cathode materials for electrochemical device applications.

## 1. Introduction

The increasing demand for global energy, which has an important influence for energy security, environmental conservation, and human health, has brought intensive attention to the exploration of alternative energy conversion and storage devices with the features of high efficiency, low cost, and environmental friendliness [1,2,3]. Therefore, it is imperative to explore heterogeneous catalysts or electrode materials to substantially reduce production wastes and improve reaction activity at the same time [4]. In view of this, many novel materials with enhanced energy conversion and storage, such as heteroatom-doped carbons [5], nanoscale metal oxides, and conjugated polymers [6], have been widely studied in electrochemical devices [7]. Discovery of heteroatom-doped carbon materials has provided an eminent alternative to electrode materials, since conventional carbon materials (e.g., carbon blacks, nanotubes, and fibers) usually provide only limited electrochemical activities [8,9]. It should be noted that the electronic and geometric properties of carbon materials can be modulated by doping heteroatoms that can provide more active sites and improve the interaction between carbon frameworks and active sites [10,11]. The introduction of nitrogen atoms plays a more critical role in modifying the carbon frameworks as compared with other heteroatoms (such as B, P, and S), because nitrogen and carbon atoms have comparable atomic size and five valence electrons, the nitrogen atoms can form strong covalent bonds with carbon atoms [12,13]. Compared to those undoped counterparts, nitrogen-doped carbon materials with good electronic conductivity have been experimentally determined to be more compartmentalized and disordered, which can not only serve as active sites, but also guarantee the superior electrochemical performance of some energy devices [14,15,16]. For example, nitrogen-doped carbon materials with numerous extrinsic defects and active sites have been determined to improve the capacitance via surface Faradaic reactions [17,18].

In recent years, plentiful review papers concerning carbon-based nanomaterials for energy storage, including supercapacitors and electrocatalysis (such as the oxygen reduction reaction, ORR), have been reported. For example, in 2013, Paraknowitsch and coworkers highlighted the development of advanced doped carbon materials with heteroatoms beyond nitrogen. Doped carbon materials with boron, sulfur, and phosphorus, and their applications in electrochemical devices, were discussed. In this paper, the authors mentioned that boron, with the same role as nitrogen, can change the electronic structures of carbon materials in different ways, and synergistic effects by using both dopants could be realized [19]. Li et al. reviewed the development of nanocarbon ORR electrocatalysts in alkaline media and their potential applications in anion exchange membrane fuel cells and metal-air batteries. The authors suggested that it would be crucial to develop an electrode layer to enhance the activity viable for practical devices, which could introduce the high-performance non-precious metal catalysts into alkaline fuel cells and metal-air batteries. Additionally, the ionic/electronic conductivity and oxygen/product mass transport within the catalyst layers could be maximized by fabricating cathodes with an optimized microstructure and morphology [20]. Recently, reasonable design and preparation of noble-metal-free ORR electrocatalysts (such as heteroatom-doped carbon nanomaterials, transition metal-based nanoparticle-carbon nanocomposites, and iron carbide-based nanoparticle-carbon nanocomposites), with respect to the engineering of advanced carbon nanomaterials with different dimensions, have been summarized by Guo et al. The authors mentioned that the surface area and stability of the electrocatalyst for long-term operation could be enhanced simultaneously by introducing advanced carbon nanomaterials into the noble-metal-free ORR catalysts [7]. Notably, reviews focusing on heteroatom-doped carbon structures derived from conjugated polymers (CPs) are relatively scarce.

CPs, such as polyaniline (PANI), polypyrrole (PPy), polythiophene (PTh), and their derivatives, usually possess highly π-conjugated polymeric chains [21,22]. The chemical or electrochemical redox reactions can directly adjust the conductivity of CPs in a wide range between 10^−10^ and 10^4^ S∙cm^−1^ [23], endowing CPs as insulators, semiconductors, or conductors [24,25]. In addition, extensive device applications of CPs (e.g., energy storage and conversion devices, sensors, and actuators) are based on the doping, which is a reversible process and makes its backbone positively- (p-doping) or negatively-charged (n-doping). The rationally-designed nanostructured CPs can provide some extraordinary features, including tunable conductivity, flexibility, and mixed conductive mechanisms with the ability to decrease the interfacial impedance between electrodes and electrolytes [26,27,28]. Along with the intensive studies on carbon materials in the last few decades, heteroatom-doped carbon materials derived from CPs can be considered as new but significant members with the promising application in energy devices, because these CPs contain only nonmetallic elements and some pivotal heteroatoms in their backbones, providing unique opportunities for the synthesis of metal-free heteroatom-doped carbon materials by direct carbonization of the CPs [29,30]. The heteroatoms homogeneously introduced into the carbon framework can not only be preserved at a relatively high content by adjusting the carbonization temperature, but also stay stable under harsh working conditions [31,32]. Direct carbonization of the CPs avoids harsh conditions required by conventional chemical vapor deposition, thermal annealing with the presence of NH_3_, nitrogen plasma treatment, and the arc-discharge method. With the rapid development of CP-derived heteroatom carbon materials, a systematic review will be of great importance to understand the recent advances and future prospects. This review focuses on the most recent development of advanced heteroatom-doped carbon nanomaterials derived from CPs on the rational design, synthesis, and their applications as high-efficiency catalysts and electrode materials. Additionally, the challenges of fabricating CP-derived heteroatom-doped carbon materials with large-scale production, controllability of doping configurations, and excellent electrochemical performance are discussed. We hope this review can provide a profile of synthesizing functional heteroatom-doped carbon nanomaterials through the CP-derived technique, as an important alternative to the conventional routes.

## 2. Carbon Nanomaterials Derived from PANI

PANI, with aromatic rings connected via nitrogen-containing groups, is regarded as a promising template compound for the synthesis of nitrogen-doped carbon materials. Due to the similarity in the structures, incorporation of nitrogen-containing active sites into the carbon matrix can be facilitated by using PANI as a nitrogen precursor, which can also promise a homogeneous distribution of nitrogen sites and an increase in the active-site density on the surface of carbon materials [33]. In this regard, PANI can act as both the carbon source and nitrogen precursor, leading to the formation of nitrogen-containing sites homogeneously distributed on the graphitic carbon surface during the catalyst synthesis.

### 2.1. Catalytic ORR Activity of PANI-Derived Nitrogen-Doped Carbon Nanomaterials

**Figure 1:**
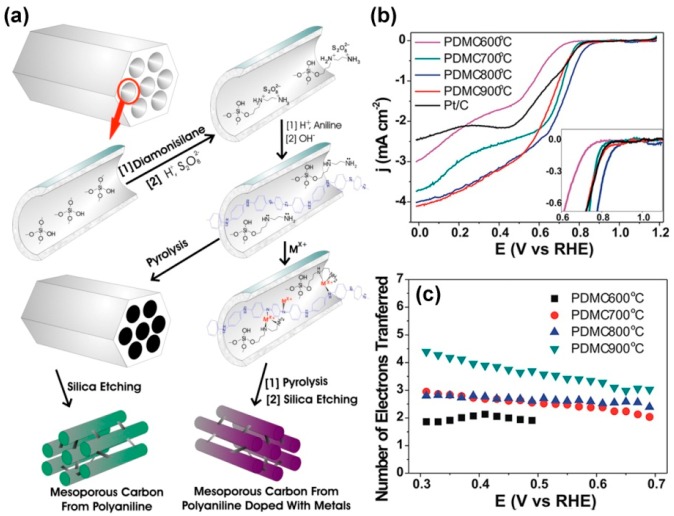
(**a**) Fabrication of N- and O-doped mesoporous carbons by calcination of PANI/SBA-15 and then etching SBA-15. Linear sweep voltammetry (LSV) curves at 900 rpm (**b**) and the number of electrons transferred as a function of potential for PDMCs prepared at different temperatures (**c**). Reprinted with permission from [35].

By now, a variety of nitrogen-doped carbon nanomaterials derived from PANI with excellent ORR performance have been developed, such as pyridinic- and pyrrolic-nitrogen-doped graphene [34], N- and O-doped mesoporous carbon materials [35], morphology-controlled nitrogen-doped carbon materials [36], nitrogen-doped carbon nanospheres [37], and so on [38,39]. For example, Silva and co-workers reported the O- and N-doped mesoporous carbon by carbonization of the in situ polymerized mesoporous silica-supported PANI, followed by etching away the mesoporous silica template (Figure 1a) [35]. From the rotating disk electrode (RDE) results, the catalytic performance of the PANI-derived mesoporous carbons (PDMCs) as metal-free electrocatalysts for ORR is directly dependent on the pyrolysis temperature, where PDMC-800 presents the best activities with the lowest overpotential toward ORR in O_2_-saturation 0.1 M KOH solution, while PDMC-900 features a one-step, four-electron pathway (Figure 1b,c). According to X-ray photoelectron spectroscopy (XPS) results, co-existence of N and O species on the PDMC and adduct formation between dioxygen and the pyridine groups in the PDMCs would be responsible for the higher ORR catalytic activity. In addition, PANI-derived N and other heteroatoms (e.g., B, S, and P, etc.) co-doped carbon materials could provide improved performances toward ORR because of their increased asymmetrical atomic spin density [6,10,40]. Recently, Dai et al. fabricated three-dimensional N and P co-doped mesoporous nanocarbon (NPMC) foams through a template-free method, simply by pyrolysis of PANI aerogels with the presence of phytic acid [41]. NPMC catalysts had a large surface area of ~1663 m^2^·g^−1^ and good electrocatalytic properties for both ORR and oxygen evolution reaction (OER), with a positive onset potential of 0.94 V vs. reversible hydrogen electrode (RHE) and half-wave potential of 0.85 V vs. RHE that are comparable to those using Pt/C catalyst in O_2_-saturated 0.1 M KOH solution.

**Figure 2:**
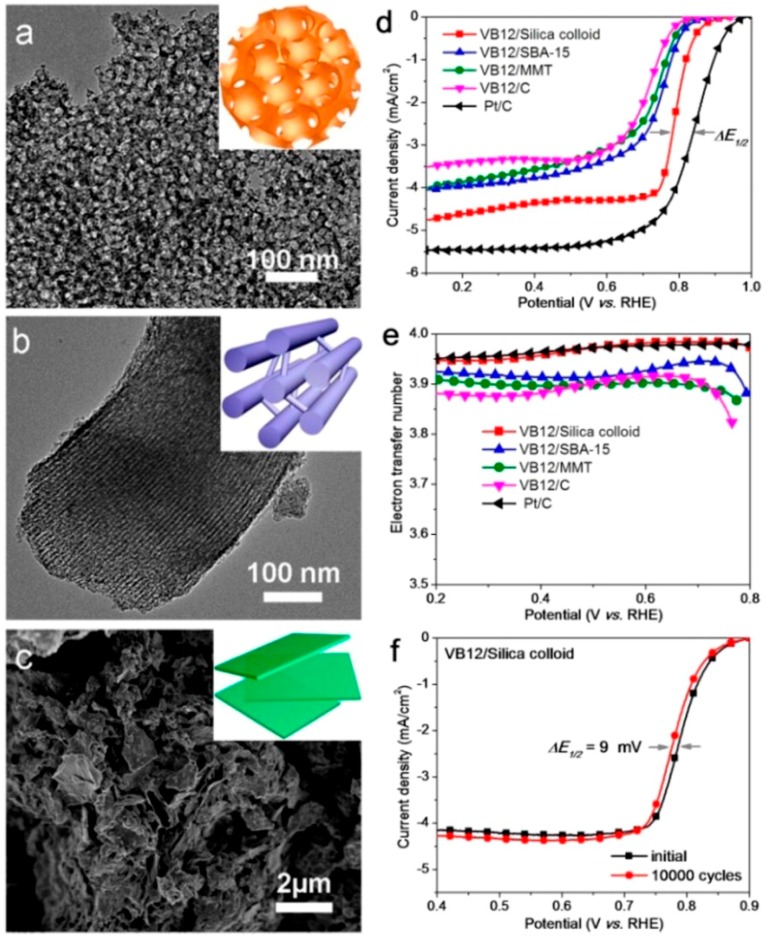
(**a**,**b**) TEM and (**c**) SEM images of fabricated C–N–Co catalysts: (**a**) VB12/silica colloid; (**b**) VB12/SBA-15; and (**c**) VB12/montmorillonite (MMT). Insets of (**a**–**c**) are the model illustration of the materials with multiple mesoporous structures. LVS polarization plots at 1600 rpm, 10 mV/s (**d**) and electron-transfer numbers (**e**) of different C–N–Co materials and Pt/C as functions of the electrode potential; (**f**) LSV polarization curves of VB12/silica colloid before and after 10,000 potential cycles in O_2_-saturated electrolyte. Reprinted with permission from [45].

Another effective method to enhance the catalytic performance of nitrogen-doped carbon is to introduce transition metal (Fe, Co, Mn) active centers [7,42]. The incorporation of transition metals could not only prompt the formation of metal-nitrogen (M-N) active sites, but also catalyze the carbonization reaction [43,44]. The ORR performance of N, M co-doped carbon catalysts strongly relies on the type of nitrogen and transition-metal precursors, pyrolysis temperature, and synthesis conditions. Wu et al. reported a PANI-M-C catalyst through an approach of using PANI as a carbon-nitrogen template to incorporate Fe and Co [33]. The resulting PANI-Fe-C materials were employed as ORR catalysts, which exhibited good electrocatalytic activity, remarkable performance stability, and excellent four-electron selectivity (hydrogen peroxide yield < 1.0%) in O_2_-saturated 0.5 M H_2_SO_4_ solution. Using silica nanoparticles, ordered mesoporous silica SBA-15, and montmorillonite as templates, Feng et al. fabricated metal-nitrogen-doped carbon electrocatalysts [45]. As reported, vitamin B12 (VB12) and PANI-Fe complex were the best precursors to prepare the C–N–Co and C–N–Fe mesoporous nonprecious metal (NMP) catalysts, respectively. From the TEM and SEM images, one could see various morphologies, including interconnected vesicle-like frameworks, linear arrays of mesoporous structures, and nanosheet structures (Figure 2a–c). In view of their well-defined porous structures with a narrow mesopore size distribution, high Brunauer-EmmettTeller (BET) surface area, and homogeneous distribution of abundant metal-N*_x_* active sties, the mesoporous catalyst prepared from VB12 and silica nanoparticles could be highly active in acidic conditions, with a half-wave potential of 0.79 V vs. RHE, high selectivity (electron-transfer number > 3.95), and promising electrochemical stability (half-wave potential negatively shifted by only 9 mV after 10,000 cycles), as shown in Figure 2d–f. Furthermore, nitrogen-doped carbon catalysts were also fabricated by pyrolysis of the composite prepared through the polymerization of aniline with PVP both as the soft template and stabilizing reagent [46], which showed that the high catalytic activity, excellent methanol and SO_2_ tolerance, and superior stability in acid electrolyte could be attributed to the textural structure and pore structure of the catalysts.

### 2.2. Electrode Material of PANI-Derived Nitrogen-Doped Carbon Nanomaterials

The nitrogen-doped carbon nanomaterials (NDCMs) derived from PANI not only are considered as an excellent candidate for ORR, but also show enhanced capacitance from the synergetic effect of both double-layer and faradaic features [47,48]. It is noted that the capacity for energy storage of NDCMs derived from PANI strongly depends on the heat-treatment temperature and synthetic methods. For example, Yang et al. reported nitrogen-doped carbon nanotubes (NCNTs) through direct carbonization of PANI nanotubes, and found that NCNTs containing appropriate content of nitrogen obtained at 700 °C exhibited high specific capacitance (163 F∙g^−1^) at a current density of 0.1 A∙g^−1^ and superior rate capability in 30 wt % KOH aqueous solution [49]. Using PANI nanowires as a carbon precursor, Yuan et al. fabricated nitrogen-enriched carbon nanowires by the direct carbonization method at different temperatures [50]. The materials synthesized at 700 °C gave the highest specific capacitance (327 F∙g^−1^) at 0.1 A∙g^−1^, excellent stability, and reversibility, due to its higher mesoporous ratio and appropriate nitrogen content. Peng et al. also developed nitrogen-containing PANI-based carbon nanospheres (C-PANI) via direct carbonizing PANI nanospheres at different temperatures [51]. The C-PANI-800 with 6.69% nitrogen content obtained at 800 °C presented high capacitance of 359 F∙g^−1^ at 1 A∙g^−1^, excellent rate capability (81% retention at 20 A∙g^−1^), and superior cyclic stability in alkaline medium.

**Figure 3:**
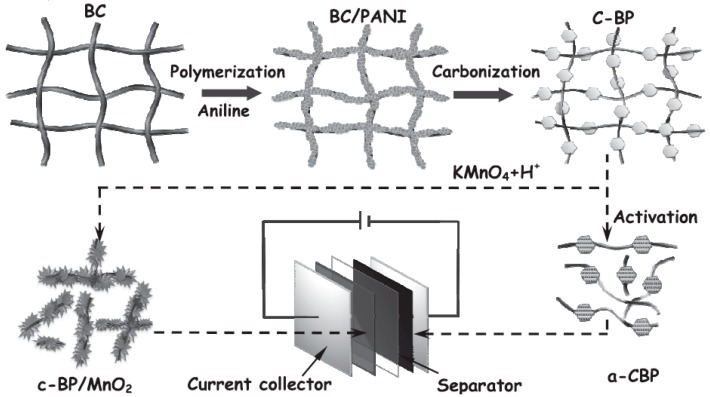
Schematic illustration for the fabrication of the electrode material application in an asymmetric supercapacitor device using bacterial cellulose (BC) as a template and precursor. Reprinted with permission from [54], copyright 2014, Wiley-VCH.

Likewise, the capacitance of NDCMs is also influenced by the synthesis techniques. For instance, Tan et al. developed a novel template method to synthesize ultrathin nitrogen-doped graphitic carbon nanocages (CNCs), which was realized by carbonization of the hydrothermally obtained Mn_3_O_4_@PANI core-shell nanoparticles and subsequent removal of the MnO cores by acidic treatment [52]. Under the optimal carbonization temperature of 800 °C, the prepared CNCs-800 exhibited regular frameworks, high specific surface area, and appropriate N content, providing high specific capacitance (248 F∙g^−1^), excellent rate capability, and outstanding cycling stability in 6 M KOH solution. Han et al. prepared hollow PANI spheres (HPS) using sulfonated polystyrene spheres (SPS) as a hard template and obtained porous nitrogen-doped hollow carbon spheres with enhanced electrochemical performance by carbonizing HPS [53]. Additionally, with PANI-coated bacterial cellulose (BC) as both template and precursor, Long et al. synthesized nitrogen-doped activated carbon (a-CBP) and carbon-MnO_2_ hybrid materials (c-BP/MnO_2_) (Figure 3) [54]. The materials exhibited high energy density (63 Wh∙kg^−1^) and excellent cycling performance when a-CBP and c-BP/MnO_2_ were used as the negative and positive electrode materials for an asymmetric supercapacitor (ASC) device.

## 3. Carbon Nanomaterials Derived from PPy

### 3.1. Catalytic ORR Activity of PPy-Derived Nitrogen-Doped Carbon Nanomaterials

**Figure 4:**
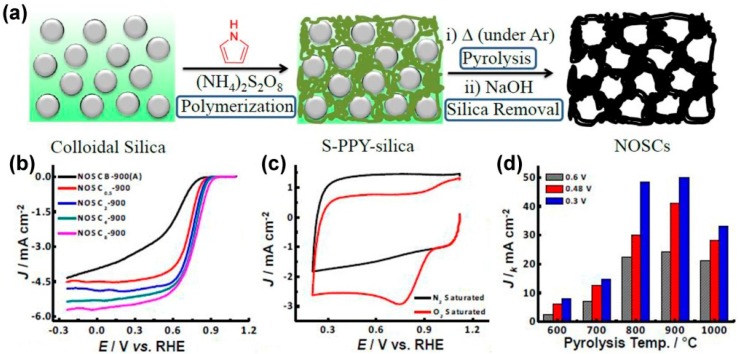
(**a**) Fabrication of N-, O-, and S-tridoped mesoporous carbon (NOSCs) with the ability of catalyzing ORR and AOR; (**b**) ORR polarization plots of NOSC_x_-900 materials on RDE rotating at 1600 rpm; (**c**) CV curves of NOSC_8_-900 in 0.1 M KOH conditions with O_2_ and N_2_-saturated; and (**d**) kinetic current density (*J*_k_) of NOSC_8_-T catalysts for ORR at different potentials. Reprinted with permission from [58].

**Figure 5:**
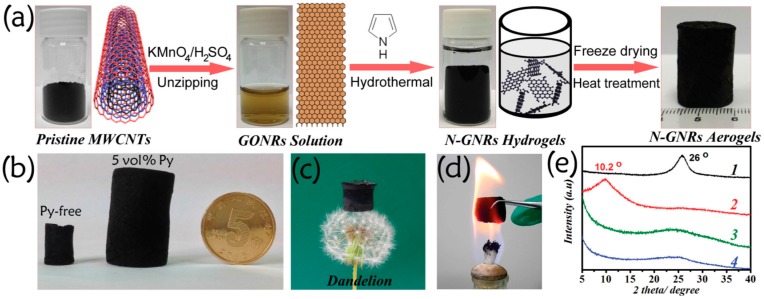
(**a**) Illustration of the synthesis for N-GNRs-A; (**b**) photographs of fabricated 3D graphene nanoribbon aerogels derived without (left) or with (middle) 5 vol % Py GONRs suspension (10 mg∙mL^−1^) under hydrothermal treatment. Photograph of ultralight N-GNRs-A standing on a dandelion (**c**) and in a hot flame of an alcohol burner (**d**); and (**e**) XRD patterns of (1) pristine MWCNTs, (2) GONRs, (3) GNRs-A, and (4) N-GNRs-A. Reprinted with permission from [62], copyright 2015, Wiley-VCH.

Though NDCMs as ORR catalysts have been widely studied, it is important to know that controlling the structure or morphology is crucial to improve the catalytic activity of carbonized polymer catalysts [55,56]. Morozan et al. synthesized PPy with granular-and tubule-like morphologies and studied the relationship between the morphology and ORR performances of the PPy-derived carbon materials [57]. The annealed PPy with tubule-like morphology exhibited higher ORR performance, higher current density, and higher electron transfer number. As mentioned above, a new synthetic method is indispensable for obtaining heteroatom-doped carbon materials with better structures. At this juncture, the most popular method is nanocasting, which uses porous materials as templates. Meng et al. reported metal-free N-, O-, and S-tridoped mesoporous carbons (NOSCs) using colloidal silica nanoparticles as templates through the carbonization of S-containing PPy/silica composite materials and subsequent removal of the silica templates (Figure 4a) [58]. From the LSV and CV curves, NOSC_8_-900 (the amount of colloidal silica is 8 g) exhibited the most positive onset potential (~0.96 V vs. RHE), the largest current density, and the highest kinetic current density, indicating its highest ORR electrocatalytic activity (Figure 4b–d). It was pointed out that more C–S–C type sulfur, and pyrrolic and quaternary nitrogen species were formed in NOSC_8_-900, which resulted in excellent electrochemical performance towards ORR. Controllable structure and morphology of heteroatom-doped carbon materials are always deemed as the key points to improve their ORR performance [59]. Recent reports suggested that graphene nanoribbons (GNRs) with high aspect ratios, straight edges, and few defects on the basal plane would be more suitable as functional materials [60]. Moreover, the electronic energy gap of GNRs can be modulated by heteroatom doping, which is beneficial for enhancing their electrocatalytic activity [61]. In view of this, as shown in Figure 5, nitrogen-doped GNR aerogel (N-GNRs-A) with high conductivity (0.5–35.6 S∙m^−1^), ultralight, neat, and versatile properties were fabricated by a facile hydrothermal method using pyrrole (5 vol %) as the nitrogen source [62]. Pyrrole monomer absorbed on the surface of graphene oxide nanoribbons through hydrogen bonding and π-π interactions not only acted as the nitrogen source, but would also guarantee the quality of the gel (the hydrogel would not shrink when the amount of pyrrole monomer was 5 vol %). The as-prepared N-GNRs-A catalyst exhibited highly positive onset potential, high cathodic current density, and promising durability for ORR as compared to the commercial Pt/C catalysts in an alkaline medium. Additionally, the N-GNRs-A catalysts also had an excellent ORR activity in acidic conditions. The authors also suggested that the aerogel technique might provide a new way for the preparation of high-performance GNR-based porous materials for energy device applications. They also developed carbon nanoleaves with three-dimensional porous aerogel networks as electrocatalysts for ORR, which showed ultrafast and sufficient mass transfer and restrained the aggregation of adjacent CNTs or GNRs. Compared with commercial Pt/C catalysts, this network structure presented a more positive onset potential, very limited hydrogen peroxide production, and better durability in both acidic and alkaline media [63].

Likely, transition metal-nitrogen-carbon (M-N-C) materials have been considered as the best non-precious metal alternative catalysts for ORR. The O_2_ adsorption and reduction can be improved by the introduction of M and N atoms because they could induce an uneven charge distribution [64,65]. PPy, containing abundant pyrrole-type nitrogen, has been widely used for synthesizing M-N-C catalysts. Feng et al. proposed that introduction of sulfur as a dopant could further improve the electrocatalytic activity of M-N-C materials due to the formation of extended graphene layers, which could offer more active sites and better interactions between the catalytic sites and the surrounded carbon in the carbon matrix, and Co-PPy/C doped by different sulfo-groups were prepared [66]. Their results showed that the surface concentrations of nitrogen and cobalt were increased by the addition of dopants, resulting in the formation of more active sites for the ORR. One of the critical issues is the selection of the sulfur source. With sodium dodecylbezene sulfonate (DBSNa), sodium paratoluene sulfonate (TSNa), and sodium benzene sulfonate (BSNa) as the sulfur sources, it was confirmed that the BSNa-doped catalyst presented the best ORR activity. In addition, nitrogen/sulfur co-doped non-precious metal materials using p-toluenesulfonic acid (TsOH) as another sulfur source as a platinum-free catalysts for ORR had also been developed [40]. Comparing with the TsOH-free catalyst, the catalysts doped with TsOH exhibited significantly enhanced ORR performance with a four-electron process, exceptional stability, and higher methanol tolerance. Yuan et al. developed Co-PPy-TsOH/C catalysts with superior ORR performance, also using TsOH as the sulfur source [67]. The analytical method for characterizing nitrogen sites in M-N-C materials is usually X-ray photoelectron spectroscopy (XPS). Kuroki et al. reported a new method to analyze nitrogen sites in nitrogen-doped carbon catalysts by employing ^15^N solid-state NMR [68]. It was found that there were pyridinic, quaternary, and pyrrolic nitrogen in carbonized PPy, and the iron-containing carbonized PPy catalysts showed improved ORR activity. Other M-N-C catalysts derived from PPy have also been developed in the last decades [69,70].

The most universal technique to synthesize mesoporous carbon materials with high specific surface area and unique ORR performance is the nanocasting method, using mesoporous silica as the hard template. The silica template must be removed in this process, which would be time-consuming and need dangerous hydrofluoric acid treatment. In order to overcome this defect, Wan et al. developed a novel impregnation method or vaporization-capillary condensation method to fabricate nitrogen-doped ordered mesoporous carbon materials [71]. It was found that the prepared carbon materials showed excellent ORR activity and two times higher performance than Pt in methanol fuel cell test. Very recently, Meng et al. developed Fe–N-doped mesoporous carbon microspheres (Fe–NMCSs) using mesoporous Fe_3_O_4_ microspheres as a source of Fe and an oxidation agent for the polymerization of pyrrole [72]. This technique not only successfully avoided the danger of HF or hot alkaline treatment, as in conventional methods, but also enhanced the utilization and digestion of Fe_3_O_4_ templates. The Fe–NMCSs with a diameter of 250 nm possessed abundant small pores over the microspheres among the interconnected small particles. The obtained Fe–NMCSs catalyst presented a superior ORR performance in comparison to commercial Pt/C in both alkaline and acidic media. They also mentioned that the Fe–NMCSs could triumphantly serve as a promising cathode catalyst both in real alkaline and proton fuel cells.

### 3.2. Electrode Material of PPy-Derived Nitrogen-Doped Carbon Nanomaterials

**Figure 6:**
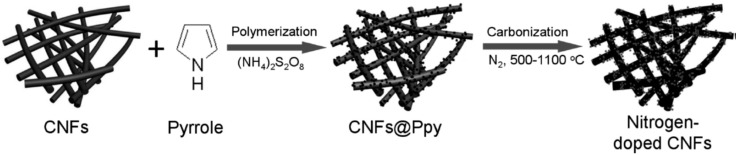
Schematic illustration for the fabrication processes of nitrogen-doped CNFs. Reprinted with permission from [75].

One approach to further enhancing the energy density and specific power of capacitors is introduction of heteroatoms into the carbon frameworks with pseudocapacitance properties because the activity and electric conductivity of carbon materials can be enhanced by the presence of heteroatoms [73,74]. The most effective method to introduce nitrogen into carbon frameworks is using nitrogen-containing materials as precursors, with the possibility to incorporate relatively high nitrogen content, and the nitrogen could stay stable under harsh working conditions. In view of this, Chen et al. developed high-content nitrogen-doped porous carbonaceous nanofibers (CNFs) containing graphitic frameworks via carbonizing macroscopic-scale CNFs coated with PPy at an appropriate temperature (Figure 6) [75]. Compared with the most popular nitrogen-containing carbon materials as electrode materials for supercapacitors, CNFs displayed promising electrochemical performance with a high specific capacity (202.0 F∙g^−1^ at a current density of 1.0 A∙g^−1^), cycling stability, and a maximum power density of 89.57 kW∙kg^−1^. The facts that nitrogen incorporation can improve the wettability of carbon for electrolyte accessibility and adjust pore texture to form electric double-layer capacitance are essential for nitrogen-doped carbon as the electrode materials in supercapacitors. An et al. prepared nitrogen-doped carbon layer-coated carbon nanotubes (NC-CNTs, 8.6 at% nitrogen content) with larger specific surface area and hierarchical pore structure through a simple two-step procedure: in situ growth of PPy onto CNTs and subsequent carbonization of the PPy/CNTs hybrids [76]. In 6 M KOH, NC-CNTs exhibited much higher capacitance (205 F∙g^−1^) and superior cycling stability due to the synergetic effect of abundant nitrogen containing groups and improved pore structure. As mentioned above, porous carbon has attracted increasing interest in many fields because of its high specific surface area, excellent electrical conductivity, and environmental friendliness [77,78]. However, one of the most efficient and typical methods to obtain porous carbon with high surface area is based on KOH activation via pyrolysis of the mixture of carbon containing species and KOH at high temperature. Hence, by selecting PPy microsheets as carbon and nitrogen precursors and KOH as the activating agent, Qie et al. developed functionalized 3D hierarchical porous carbon (THPC) materials with very high specific surface area (2870 m^2^∙g^−1^), high content of doped heteroatom (N: 7.7 wt %, O: 12.4 wt %), excellent electrical conductivity, and hierarchical porous structure by a facile modified chemical activation route [79]. In addition, the prepared THPC showed excellent rate performance, high capacitance, and long-term stability as the electrode material for supercapacitors in both aqueous and organic electrolytes due to the unique features. Additionally, nitrogen-containing carbon nanotubes (NCNTs), by the carbonization of CPs with nanotubular morphology, used in energy conversion and storage, sensor technology, and catalysis, have also been intensively studied due to their highly versatile applicability. PPy-derived nitrogen-doped carbon nanotubes with better electrochemical properties have been reported [80]. In order to enhance the electrical conductivity and decrease ion-transport resistance and diffusion distance, one of the most effective methods is to integrate with other conductive reagents or use activated nitrogen-doped graphene, which could further improve the energy density and power density of the supercapacitors [81].

## 4. Carbon Nanomaterials Derived from Derivatives of CPs

**Figure 7:**
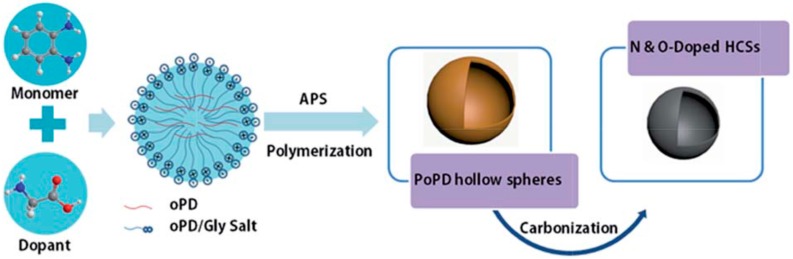
A schematic illustration depicting the synthesis route for N- and O-doped HCSs. Reprinted with permission from [85], copyright 2015, RSC Publishing.

Recently, CP derivatives (such as poly(*o*-phenylenediamine), PoPD, and poly(*o*-methylaniline), POT) with the presence of substituent groups have provided more versatility than conventional monoamine polymers (e.g., PANI, PPy). PoPD and the derived carbon materials also have potential applications in electronic devices such as fuel cells and supercapacitors [82]. As electrode materials for supercapacitors, PoPD can be chosen as a nitrogen-rich carbon precursor based on its aromatic diamines and high content of nitrogen, although bare PoPD shows an inconspicuous specific capacitance (16 F∙g^−1^). Zhu et al. fabricated activated carbons (ACs) through a one-step method with PoPD as precursor [83], using FeCl_3_∙6H_2_O to induce the polymerization of monomers and promote the formation of a porous framework during the carbonization process. The as-prepared carbon materials showed superior capacitive behavior (large capacitance, high energy and power density, and long cycle life), due to the synergetic effect of electrostatic accumulation and Faradic transitions. The authors also mentioned that a non-aqueous system was preferred in order to enhance the power and energy density. Peng et al. prepared three-dimensional porous nitrogen-doped carbon networks (3D N-CNWs) containing high percentages of nitrogen via the carbonization of poly(*p*-phenylenediamine) (PpPD) using the similar synthesis method to Zhu et al., namely the integration of oxidative polymerization and catalytic carbonization [84]. The 3D N-CNWs obtained at 700 °C possessed high specific surface area and exhibited the maximum specific capacitance (304 F∙g^−1^) at a current density of 0.5 A∙g^−1^ and excellent cycling performance. Similarly, in order to obtain N- and O-doped hollow carbon spheres (HCSs) with enlarged specific surface area due to the inherent electrical properties, enhanced electron, or ion transfer and capacitance, Yuan et al. directly carbonized PoPD submicrospheres with the assistance of glycine dopant (Figure 7) [85]. The mesoporous HCSs displayed high specific surface area (~355 m^2^∙g^−1^), high specific capacitance (210 F∙g^−1^), and decent cycling stability.

**Figure 8:**
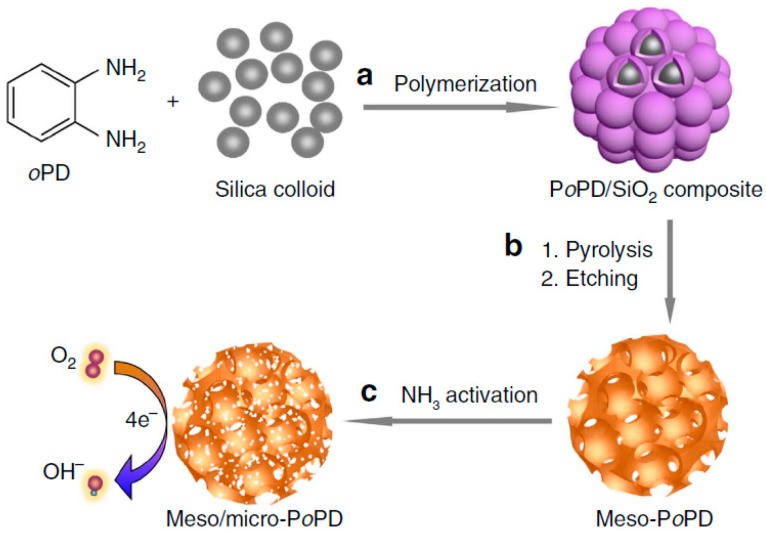
Schematic illustration of the fabrication of meso/micro-PoPD electrocatalyst. (**a**) Polymerization of oPD using the silica colloid as templates, and with the assistance of ammonium peroxydisulphate (APS); (**b**) carbonization of PoPD/SiO_2_ materials in N_2_ atmosphere, followed by etching the SiO_2_ templates with 2.0 M NaOH solution to yield meso-PoPD; (**c**) the formation of the final meso/micro-PoPD materials by the further activation of the meso-PoPD with NH_3_. Reprinted with permission from [87].

In the process of fabricating nitrogen-doped carbon materials derived from PoPD, the ladder aromatic structure of PoPD would be transformed into a polycyclic-type structure, where nitrogen atoms could be successfully incorporated into the graphitic carbon structures. These samples could exhibit much higher electrocatalytic performance for ORR and better capacitive behavior than multi-walled carbon nanotubes and nitrogen-doped carbon nanotubes [86]. To obtain metal-free catalysts competitive with Pt/C, the surface functionalities (mainly referring to the content and type of the heteroatoms) that determine the intrinsic nature of active sites, specific surface area, and porous structure need to be considered systematically. It is believed that the nitrogen types and ratios, and hierarchically porous structure which could supply numerous accessible active sites are favorable for the efficient mass transport in the catalyst layer. Liang et al. developed nitrogen-doped metal-free ORR catalysts using hard-templating method, with nitrogen-rich PoPD as the precursor [87]. The formation of hierarchically porous structures and surface functionalities of the catalysts was ascribed to the NH_3_ activation and the specific surface area of final catalysts was 1280 m^2^∙g^−1^. The excellent ORR activity with half-wave potential of 0.85 V vs. RHE in alkaline media was due to the mesoporous/microporous structure and desired nitrogen doping of the as-prepared catalysts (Figure 8). It is noted that the location of the two amino groups in aromatic ring plays a key role in determining the ORR performance of the catalysts, especially the non-precious Fe/N co-modified carbon electrocatalysts. In order to elucidate the relationship between the actual structure and performance in M-N-C catalysts derived from poly-phenylenediamine, Zhu et al. developed three Fe/N-containing catalysts based on polymerized *o*,*m*,*p*-phenylenediamine, termed as PoPD–Fe–C, PmPD–Fe–C, and PpPD–Fe–C, respectively [88]. PpPD with the smallest particle size (~1 μm) could not only facilitate the iron atom diffusion that is essential for generating ORR active Fe–N complexes, but also result in higher iron contents in PpPD–Fe–C electrocatalysts. XPS results demonstrated that Fe species in all three samples was N–Fe bonds with the highest quantity in PpPD–Fe–C catalysts. The different N–Fe contents could root in the different polymerization and surface states of the polymerized o,m,p-phenylenediamine isomers, and the real active sites for ORR were determined to be FeN_6_. Moreover, PpPD–Fe–C could also serve as a non-precious metal catalyst for ORR, which exhibited the best performance and good durability.

**Figure 9:**
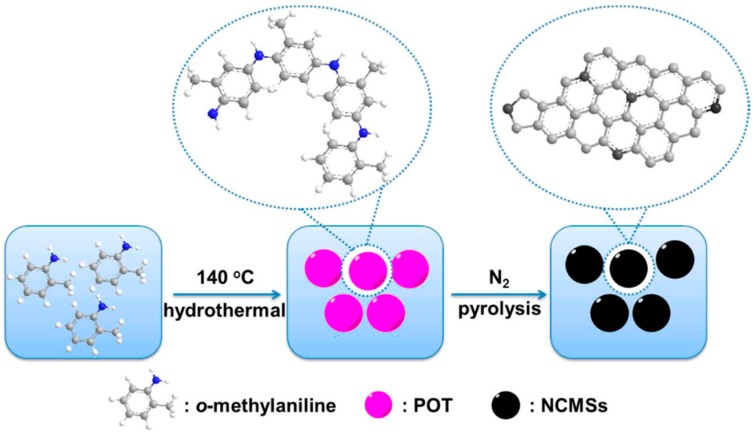
Schematic illustration of the preparation of poly(*o*-methylaniline) (POT) by a hydrothermal technique and N-doped carbon microspheres (NCMSs) from pyrolysis of POT. Reprinted with permission from [89].

The physicochemical properties (e.g., solubility, crystallinity, and electrical conductivity) of poly(*o*-methylaniline) (POT), a kind of CP derivative, could be improved by the presence of a methyl group. Our recent work reported a metal-free microporous nitrogen-doped carbon microspheres (NCMSs) catalyst through a direct carbonization of POT microspheres obtained by a facile hydrothermal method (Figure 9) [89]. The resultant carbon materials preserved the structural integrity, monodispersity, and spherical morphology with a negligible size change after high-temperature treatment, which could be attributed to the presence of a methyl group with the function of affecting the molecular rearrangement and sterical hindrance during the doping process. The reason of selecting microspheres was their great practical application due to the extraordinary building blocks for multifunctional hybrid materials. The NCMSs-900 (obtained by 900 °C carbonization) exhibited high selectivity and long stability as a metal-free catalyst for ORR and high specific capacitance because of its highest surface area and quaternary N content. Similarly, M-N-C catalysts could be obtained from the combination of POT and transition metal, such as POT-Co-ordered mesoporous carbon (OMC) composite, which displayed a four-electron pathway in alkaline electrolytes and preferable anti-crossover to methanol oxidation in ORR catalysis [90].

Another significant application of heteroatom-doped carbon materials derived from CPs is as anode materials for lithium ion batteries (LIBs), which have been extensively used in portable electronic devices because of their high energy density and long cycle life [91]. To date, carboneous nanomaterials have been reported to create more active sites for Li storage. Among them, one of the most popular materials are one-dimensional heteroatom-doped carbon nanofibers because of their superior mechanical, electrical, and structural properties [92]. Nanostructured porous carbon with surface modification with heteroatoms (N and B elements) possesses high Li^+^ ion storage capacity, reactivity, and electrical conductivity, and excellent cycling stability since the porous nanostructures can effectively shorten the Li^+^ ion transport length and provide efficient electrode/electrolyte interface for charge transfer. Qie et al. reported a porous carbon with high surface area (2381 m^2^∙g^−1^) and high-level nitrogen doping by selecting PPy nanofiber webs as a precursor and KOH as the activating agent [93]. The obtained carbon nanofiber webs (CNFWs) with nanostructures and high-level N doping possessed higher active surface area for Li^+^ ion storage as compared to the granular carbons prepared with granular PPy as precursor. The CNFWs exhibited super-high specific capability (943 mAh∙g^−1^ even at 2 A∙g^−1^ after 600 cycles), promising rate capability and cyclability as the anode material for lithium ion batteries. Other materials with higher lithium storage capacity are chiral carbonaceous nanotubes (CCNTs) which could be obtained by carbonization of self-assembled chiral PPy (CPPy) nanotubes. Che’s group proposed that chiral carbon-based nanotubes possessing helical structures and high specific surface areas would be effective one-dimensional nanomaterials owing to their improved electronic, mechanical, and thermal properties. The prepared enantiopure chiral carbonaceous nanotubes through a direct carbonization of double-helical mesoporous PPy nanotubes showed higher lithium storage [94]. These unique chiral carbon materials may have significant applications in energy storage, enantioselective separation systems, etc. The promising electrochemical energy storage performance of carbon-based materials is mainly derived from their large surface area and unique pore-size distribution. Xu et al. developed porous nitrogen-doped carbon nanotubes with high surface area and large pore volume (1.28 cm^3^∙g^−1^) via carbonizing tubular PPy, which showed high specific capacitance of 210 F∙g^−1^ at 0.5 A∙g^−1^ and excellent cycling stability as superior cathode material for lithium-sulfur batteries. To date, transition metal compounds (such as MoS_2_ and MnO_2_) are considered as promising anode materials in LIBs due to their high theoretical specific capacity, low cost, and natural abundance. However, it is the inferior rate capability and poor cycling stability that impede their practical applications in LIBs because of the particle agglomeration and low electrical conductivity. The most effective solution to address the drawbacks is the combination with carbon materials that can cushion the large stress arising from volume expansion/contraction, protect transition metal compounds from chemical corrosion and agglomeration, and increase the electrical conductivity of the hybrid materials. Guo et al. successfully synthesized rose-like MoS_2_/nitrogen-containing carbon/graphene (MoS_2_/NC/G) hybrids with boosted electronic conductivity by a facile PPy-assisted hydrothermal approach. The MoS_2_/NC/G hybrids showed improved energy storage performances (high specific capacity, excellent rate capability, and good cycling stability), as compared with bare MoS_2_ nanosheets as anode materials for LIBs [95]. Similarly, Wang et al. synthesized MnO*_x_*/pN-C (nitrogen-enriched porous carbon, pN-C) core-shell nanostructures, which exhibited superior lithium-storage performance using PPy as a nitrogen precursor through a general solution-based approach [96].

## 5. Conclusions and Outlook

In summary, this review highlighted the versatility of heteroatom-doped carbon materials derived from CPs, including PANI, PPy, and their derivatives. Meanwhile, their applications in electrocatalysis, supercapacitors, and lithium ion batteries were emphasized. High surface area, excellent mechanical properties, as well as high conductivity, are crucial to the enhanced electrochemical performance of these novel carbon materials. The nanocasting method using porous materials for preparing heteroatom-doped carbon materials can be used to improve their porosity, surface area, and electrochemical contact area by inducing hierarchical porosity. In the hybrids consisting of CP-derived heteroatom-doped carbons and transition metal compounds, the transition metal can further contribute to the catalytic performance by providing additional active sites. Notably, the presence of strong coupling effects which facilitate electron transfer between the metal and doped carbons through forming active metal-dopant sites can not only enhance the working stability of the hybrids, but also improve their electrochemical performance. Compared with noble metals, these hybrid materials can sometimes exhibit even better activity and durability.

Although significant progress has been made in the synthesis and application of heteroatom-doped carbon materials derived from CPs, challenges still exist with respect to large-scale production, control of doping configurations, and even higher electrocatalytic activity. First, a facile and green method of CP-derived heteroatom-doped carbon materials is highly desired because most of the reported fabrication techniques require high temperature and harsh conditions, making the large-scale production and commercialization difficult. Second, fine control of the composition, microstructures, and interactions between transition metal and CPs of Me-N-C hybrids is still challenging, although they usually present superior electrochemical performance due to the synergetic effects. Third, recent advances in CP-derived heteroatom-doped carbons as electrode materials are encouraging. However, these advances are at an initial stage with respect to the related studies. As a result, rational design and proper construction of heteroatom-doped carbon nanostructures derived from CPs are of importance for acquiring high performance carbon materials. Furthermore, the stability and lifetime of heteroatom-doped carbons derived from CPs are still unsatisfactory.

Therefore, CP-derived heteroatom-doped carbon materials, with excellent electrochemical activities, have potential applications in a variety of energy devices and systems. It is believed that heteroatom-doped carbon materials derived from CPs and their derivatives, with structural defects and ideal nanostructures, will have a bright future in the field of electrochemistry.
